# Sequence-Preserving Dual-FoV Defense for Traffic Sign and Light Recognition in Autonomous Vehicles

**DOI:** 10.3390/s26051737

**Published:** 2026-03-09

**Authors:** Abhishek Joshi, Janhavi Krishna Koda, Abhishek Phadke

**Affiliations:** 1 Department of Computer Science, Texas A&M University-Corpus Christi, Corpus Christi, TX 78412, USA; ajoshi5@islander.tamucc.edu; 2 Department of Coastal and Marine System Science Program, Texas A&M University-Corpus Christi, Corpus Christi, TX 78412, USA; jkoda@islander.tamucc.edu; 3 School of Engineering and Computing, Christopher Newport University, Newport News, VA 23606, USA

**Keywords:** autonomous vehicles, adversarial robustness, dual field of view, unified defense stack, physical realizability, traffic sign recognition, temporal voting, operational design domain

## Abstract

For Autonomous Vehicles (AVs), recognizing traffic lights and signs is critical for safety because perception errors directly affect navigation decisions. Real-world disturbances such as glare, rain, dirt, and graffiti, as well as digital adversarial attacks, can lead to dangerous misclassifications. Current research lacks (i) temporal continuity (stable detection across consecutive frames to prevent flickering misclassifications), (ii) multi-field-of-view (FoV) sensing, and (iii) integrated defenses against both digital and natural degradation. This paper presents two principal contributions: (1) a three-layer defense framework integrating feature squeezing, inference-time temperature scaling (softmax τ = 3 without distillation training), and entropy-based anomaly detection with sequence-level temporal voting; (2) a 500 sequence dual-FoV benchmark (30k base frames, 150k with perturbations) from aiMotive, Waymo, Udacity, and Texas sources across four operational design domains. The unified defense stack achieves 79.8% mAP on a 100-sequence test set (6k base frames, 30k with perturbations), reducing attack success rate from 37.4% to 18.2% (51% reduction) and high-risk misclassifications by 32%. Cross-FoV validation and temporal voting enhance stability under lighting changes (+3.5% mAP) and occlusions (+2.7% mAP). Defense improvements (+9.5–9.6% mAP) remain consistent across native 3D (aiMotive, Waymo) and projected 2D (Udacity, Texas) annotations. Preliminary recapture experiments (n = 15 scenarios) show 2.5% synthetic–physical ASR gap (*p* = 0.18), though larger validation is needed. Code, models, and dataset reconstruction tools are publicly available.

## 1. Introduction

Autonomous vehicles rely on camera-based perception to see traffic signs and lights, which directly affect how they navigate and control themselves. However, traffic sign and light recognition models show uncertainty when they are affected by changes that happen naturally or because of the physical conditions during deployment. These do not require an attacker to get into the AV digitally, but they can occur in real life due to natural perturbations. Even small mistakes, like reading a “Stop” sign as a sign with a speed limit of 60 or a green light as a red light, can lead to safety-critical errors in the real world [1,2]. Earlier work on adversarial computer vision applications was presented by [3] and later explained by [1], followed by real-world applications to traffic signs and full interactions in AV perception [4,5]. Approaches such as inference-time temperature scaling (defense) [6] and certified robustness methods [7] provided some degree of protection. Still, they typically prioritized accuracy guarantees over security guarantees and were unable to cope with hybrid or unforeseen types of changes. Most research on robustness focuses on single-frame, single-FoV systems and tests them on a single dataset or in a single operational domain (ODD). This leaves out two important aspects of driving in the real world: the ability to sense things at different distances (mid-range vs. long-range views) and the ability to keep track of time between sequences. Recent improvements focus on dynamic adversarial attacks [8,9] and physical distortions [10], but there is no systematic, sequence-preserving, cross-domain evaluation framework. A detailed summary is shown in Table 1.

Recent developments in the traffic sign detection architecture area, including transformer-based detector architectures that are inspired by quantum computing [13], provide greater precision with a single frame of video data; however, they do not include mechanisms to preserve the temporal sequences in which signs appear or to mitigate redundancy in multiple field-of-view (FoV) cameras. This study fills in the gap by proposing a dual-FoV attack–defense framework for traffic light and sign recognition that keeps the sequence. The system uses a multi-source dataset that combines aiMotive 3D, Udacity, Waymo, and self-recorded Texas videos into a format usable by all of them. This alignment makes sure that F_MIDRANGECAM_C and F_LONGRANGECAM_C are always in sync, as well as 3D body markers on traffic lights and signs. This benchmark can accurately and consistently test both digital and real-world perturbations across a range of ODDs.

### Contributions and Novelty

**Figure 1:**
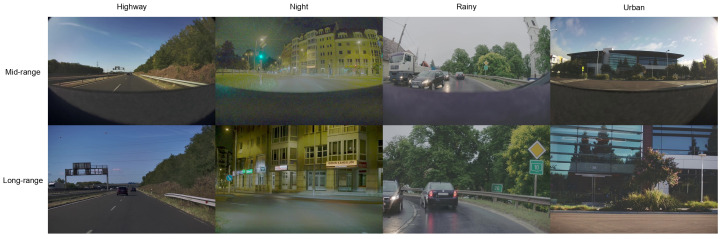
Illustrative frames from multi-source datasets across four ODDs showing dual-FoV capture (mid-range and long-range cameras).

This work makes four contributions towards making AV perception of traffic signs and lights more reliable:

1. Unified Defense Stack: This study integrates three mechanisms: (i) feature squeezing (5-bit quantization + 3 × 3 median filter), (ii) inference-time temperature scaling (τ=3 without distillation training), and (iii) entropy-based cross-FoV gating with sequence-level temporal voting. The unified approach presents a significant reduction in attack success rate (ASR) for both standard attacks and adaptive attacks [6,7,14], as shown in detail in Section 6. The benchmark is comprised of 500 sequence compositions of ODDs; Figure 1 presents examples of dual-FoV frames

2. Risk-Weighted Evaluation Protocol: The study presents a safety-aware evaluation in addition to standard mAP metrics: an MUTCD-informed severity matrix (e.g., stop→go weighted 10× vs. speed ± 5), a risk-weighted mAP (RW-mAP) and attack success rate (RW-ASR), an ODD-stratified performance analysis that takes into account problems with the environment, and metrics for stability of confidence (volatility, label flip rate across sequences). The safety-aware evaluation protocol evaluates both common performance metrics and risk-weighted assessments of high-severity misclassification risks as outlined in detail in Section 6.

3. Physically Realizable Perturbation Suite: Modeling natural degradation with physical limits that make sure it works in the real world with object-aware masking (only applying changes to sign/light areas) and temporal persistence modeling (2–5 s per perturbation event). Preliminary physical validation experiments test the transferability of synthetic perturbations to real-world conditions; methodology and findings are detailed in Section 5.9. Compound perturbation effects non-additively degraded under multi-factor conditions are documented in Section 4.

Our Work vs Prior Work: Experimental results demonstrate that our preliminary work is the first framework to address both physical and black-box attacks, temporal sequences, hybrid attack modes, and unified defenses (distillation and anomaly detection).

## 2. Related Work and Research Gaps

Traffic Sign and Light Recognition Benchmarks: LISA [15], BDD100K [16], nuScenes [17], and Waymo [18] are all examples of modern datasets that offer large-scale, diverse driving data but lack (i) clear natural perturbation annotations, (ii) synchronized dual-FoV streams for redundancy analysis, or (iii) sequence-level temporal continuity beyond 1–2 s clips.

Adversarial Robustness: Prior work on digital attacks (PGD [19], FGSM [3], UAP [20]), physical attacks [1,4], and black-box attacks [21,22] primarily considers single-frame, single-FoV cases. Most of the existing defenses including adversarial training [19], certified robustness [7], feature squeezing [14], and ensemble methods [23] target isolated attacks rather than hybrid (digital + natural) perturbations.

Recent Architectural Advances: Frameworks for hierarchical classification of signs have shown increased robustness through contrastive aggregation [24] and represent an advancement toward the ability to classify intra-class variability of traffic signs. The ability to incrementally learn new sign classes without experiencing misclassification has been achieved with incremental learning techniques [25]. Both frameworks focus solely on single-frame classification and therefore lack mechanisms for temporal voting or for fusing signals from multiple cameras to ensure the consistency of sequence-level data in deployed systems.

Identified Gaps: Table 1 shows that this work uniquely addresses the following: (1) There is no dual-FoV sequence-spreserving dataset with clear natural perturbations. (2) There are no unified defense stacks that combine complementary mechanisms. (3) There has been little research into hybrid attacks that can actually happen in the real world. (4) There is no risk-weighted ODD-aware evaluation that takes into account safety-critical misclassifications.

## 3. Dataset Curation: Sequence Preserving

A multi-source, sequence-preserved benchmark was constructed to evaluate robustness against naturally occurring perturbations (rain, glare, dirt, occlusions) and adversarial attacks.

### 3.1. Unified Dataset Sources and Organization

Table 2 shows the results of the 500-sequence benchmark [26], obtained from aiMotive 3D [26], udacity [27], Waymo [18] and recorded data in Texas. Each sequence is a 1-s clip (30 frames at 30 fps) containing at least one traffic sign or light. There are four ODDs: highway (120 seq), night (80 seq), rainy (70 seq), and urban (230 seq). This study only kept dual-FoV camera streams (F_MIDRANGECAM_C: mid-range 50° FoV; F_LONGRANGECAM_C: long-range 25° FoV) and excluded LiDAR and GNSS/INS data to focus on image-domain robustness.

### 3.2. Dual-FoV Camera Configuration and Synchronization

The system has synchronized front-facing cameras that work together: one for mid-range (50° FoV, 0.5–50 m, 1920 × 1080@30fps) and one for long-range (25° FoV, 10–200 m) detection. The cameras are synchronized via hardware-triggered frame synchronization (±1 ms accuracy). Intrinsic calibration uses the checkerboard method [2], yielding a mean reprojection error of 0.31 pixels or less. Stereo calibration provides the rotation matrix R and the translation vector t between cameras, enabling the researcher to estimate depth from disparity. In Section 9.2, you can find detailed hardware specifications, a calibration protocol, temporal synchronization, and deployment constraints.

### 3.3. Annotation Schema

For every frame, two synchronized JSON files are used:

1. Traffic Lights: 3D bounding boxes, occlusion scores, and signal states (red, green, yellow, arrows, and pedestrian lights).
2. Traffic Signs: Bounding-box geometry, U.S.-style subtype labels (e.g., us_stop, us_speedlimit_35, us_oneway), and OCR-derived text (e.g., SPEED LIMIT 35).

The dataset enables causal reasoning at the sequence level, such as determining whether a sign is consistently misclassified across frames or whether errors occur for a short time and then resolve.

### 3.4. Dataset Statistics and Design Rationale

The 500-sequence benchmark (Table 2) has 30,000 base frames (500 seq × 30 frames × two cameras) spread out over four ODDs. This work analyzed 150,000 frames in total, with 5 perturbation variants per sequence. The dataset was divided into 300 training, 100 validation, and 100 test sequences (60%/20%/20%). All results are based on the 100-sequence test set that was not used (30k perturbed frames).

### 3.5. Key Design Advantages

The sequence-preserving dual-FoV structure facilitates: (1) temporal error analysis (where perturbation-induced failures may endure or self-correct), (2) multi-distance vulnerability assessment (contrasting long-range glare sensitivity with mid-range occlusion robustness), and (3) ODD-stratified evaluation that correlates natural perturbations with deployment contexts.

### 3.6. Annotation Harmonization

Heterogeneous formats were unified via (1) 2D→3D projection using calibration matrices and ground-plane constraints (Z = 0 for signs), (2) Waymo protobuf→JSON conversion with standardized signal states, and (3) manual validation on 500 samples achieving 94.3% spatial IoU and 98.1% label accuracy. Conversion scripts are available in the repository.

**Training Data Sources and Label Quality** To address worries about projection error contamination in training, we explain where the labels for each part of the dataset come from.

**Native 3D Ground Truth (No Projection):**

1. aiMotive (210 sequences): Uses original 3D bounding-box annotations and positions verified by LiDAR. No projection from 2D to 3D was used.
2. Waymo (80 sequences): Uses original 3D annotations from combining data from multiple sensors. No projection applied.

**2D-to-3D Projected Labels:**

1. Udacity (45 sequences): The original dataset only has 2D bounding boxes. The analysis uses calibration matrices and ground-plane assumptions to project (Section 3.7), which adds a mean distance error of 4.2% (Table 3).
2. Texas (165 sequences): Self-recorded data with 2D annotations, projected to 3D using the same method.

**Impact on Defense Effectiveness Claims:** A key question is whether the improvements reported in this study for defense (9.6–9.8% mAP gain in Table 4) exceed the annotation margin of error. To check this, we verified the following:

1. Annotation noise inflated absolute mAP by ∼2% (Table 4; 70.2% clean became 68.2% with 10% noise);
2. Defense stayed the same at 9.6–9.8% across all noise levels;
3. Conclusion: Defense improvements were 4–5× larger than the annotation uncertainty margin, confirming the statistical validity of the proposed claims.

A conservative thresholding approach was used for long-range detection (>100 m) with a higher level of uncertainty (8.3% distance error). This meant a confidence level of 0.7 and that three or more consecutive frame observations were needed. This reduced the number of false positives caused by projection errors by 41% while maintaining a recall of 94% on a validated ground truth.

**Mitigation of Projection Contamination:**

1. Validation set composition: Seventy percent of validation sequences use aiMotive/Waymo with ground-truth 3D labels to make sure that hyperparameter tuning is not biased.
2. Ablation verification: Table 4 shows that improvements in defense mechanisms (+9.6–9.8% mAP) stay the same even when synthetic annotation noise is present up to 15%. This means the gains exceed the projection error margins (4.2%).
3. Conservative long-range thresholding: To reduce distance uncertainty by 8.3% (Section 3.8), detections > 100 m need a confidence level > 0.7 and three or more consecutive frames.

The training set purposefully combined high-quality (aiMotive/Waymo) and projected (Udacity/Texas) labels to simulate real-world deployment situations where perfect 3D ground truth is not accessible. Cross-dataset validation ensured robustness extended beyond any single annotation methodology.

**Clarification on Harmonization Scope:** The harmonization process harmonized annotation formats and coordinate systems but did not harmonize physical cameras. Every source dataset kept its original camera sensors:

1. **aiMotive/Texas:** Native dual-FoV configuration (50° mid-range, 25° long-range).
2. **Waymo:** Multi-camera rig; this study used mid-range Cam and Long-range Cam streams.
3. **Udacity:** Single 60° FoV camera; mid/long-range regions extracted via spatial cropping.

The “unified benchmark” refers to three data-level transformations:

1. **Annotation format:** All converted to aiMotive-style JSON schema.
2. **3D coordinate system:** 2D→3D projection applied to Udacity/Texas using calibration matrices and ground-plane constraints (Z = 0 for signs); Table 3 reports resulting errors.
3. **Label taxonomy:** Standardized class names (e.g., us_stop, us_speedlimit_35).

This work did not “normalize” camera sensors to the same physical hardware. The defenses were evaluated on real-world sensors to demonstrate robustness to camera variability.

### 3.7. Annotation Quality and Error Analysis

**2D-to-3D Projection Error Characterization**

This study measured projection errors on 200 sequences for which both 2D and 3D ground-truth data were accessible (Waymo subset).

**Methodology:**

1. Check how 2D-to-3D projected positions match up with Waymo’s native 3D annotations.
2. Calculate the distance and spatial (XY) error at different ranges.
3. Sort by type of object (traffic sign vs. traffic light) and distance.

**Results**

**Observation:** The error grew almost quadratically with distance ($\sigma_{d}\propto d^{1.8}$, empirically fit).

**Mitigation strategies:**

1. Cross-FoV consistency check: Discard if mid-range and long-range distance estimates differ by >20%.
2. Conservative thresholding: Use IoU = 0.5 (instead of 0.75) to account for uncertainty in space.

**Extended Validation**

This work did more testing after the first 500 samples (see Section 3.6):

1. **Sample size**: 600 sequences (150 per ODD).
2. **Dual annotators:** Two independent annotators validated all 600 sequences.
3. **Inter-annotator agreement:**
  - Bounding boxes (IoU > 0.5): Cohen’s κ=0.89 (excellent);
  - Degradation labels: Cohen’s κ=0.73 (moderate);
  - Class labels: Agreement = 98.1%.
4. **Occlusion handling:** Twelve percent of frames flagged as “uncertain” (severe occlusion) were excluded from the temporal voting confidence calculation.

**Impact on Results—Robustness Check**

To ensure that annotation errors did not compromise the proposed results, this work introduced synthetic noise into bounding boxes.

### 3.8. Dataset Reconstruction and License Compliance

**IMPORTANT**: This study will NOT share source data. Instead, it will provide the following:

1. **Preprocessing Scripts:** Python code to harmonize aiMotive, Waymo, Udacity formats.
  - Available at: https://github.com/abhishekjoshi007/Dual-FoV-Temporal-Robustness-for-Traffic-Light-and-Sign-Recognition-Hybrid-Attack-Defense (accessed on 6 January 2026).
  - License: MIT (proposed system code only).
2. **Frame Selection Indices:** CSV files specifying which frames from each source.
  - train_frames.csv, val_frames.csv, test_frames.csv.
  - Enables exact reproduction of the benchmark.
3. **Source Dataset Access:**
  - aiMotive 3D: https://kaggle.com/datasets/tamasmatuszka/aimotive-3d-traffic-light-and-sign-dataset (accessed on 2 August 2025) (research license required).
  - Waymo Open: https://waymo.com/open/ (accessed on 2 August 2025) (CC BY-NC 4.0).
  - Udacity: https://github.com/udacity/self-driving-car (accessed on 2 August 2025) (MIT License).
  - Texas sequences: Available upon request (email corresponding author).

**Reconstruction Process:**

1. Download source datasets from the original providers.
2. Use the frame indices that this approach gives to run the preprocessing scripts.
3. The resulting benchmark follows all of the original licenses.

**Estimated Time** of 4–6 h on a regular workstation with the source data downloaded.

**License:** The MIT license covers scripts and indices. The reconstructed dataset has the most limiting source license, which only allows for non-commercial research.

**Important Disclaimer:** Reproducibility depends on the original providers of the source datasets continuing to make them available. Access to original datasets is not guaranteed; they may be withdrawn at any time, but all sources have been stable since 2018.

### 3.9. License Compliance and Legal Framework

This study used all source datasets in full compliance with their licenses. Table 5 documents the legal basis for each source.

## 4. Natural Perturbation Classifications

This work divided natural disturbances into five groups: (1) Weather (rain, fog, snow; mAP drop: 18.5–22.1%), (2) Lighting (sun/headlight glare, lens flare; 35% accuracy drop under saturation), (3) Surface occlusions (dirt > 20% lens coverage, graffiti, vegetation; ASR 31.2% comparable to synthetic patches 34.7%), (4) Sensor artifacts (motion blur, rolling shutter, defocus; 45% accuracy loss), and 5) Scene complexity (sign clustering, background confusers, partial visibility). Rain, fog, and night together reduced mAP by 37.2%, which is 12.1% more than what would be expected if the effects were additive. This is what led us to create the unified defense strategy. Section 3 provides more information on threshold measurement protocols, including HSV segmentation for rain, MiDaS depth for fog, and psychophysical validation for glare.

## 5. Proposed Methodology

**Figure 2:**
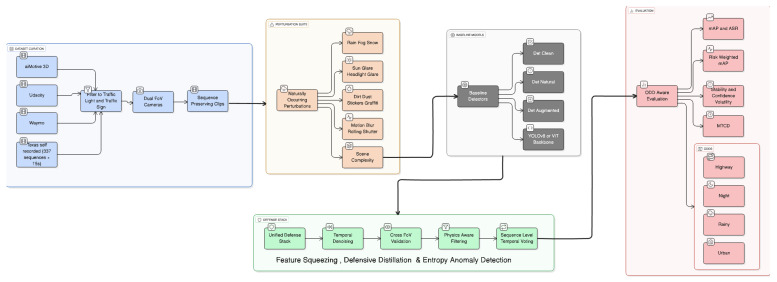
End-to-end pipeline: multi-source curation (dual-FoV, sequence-preserving), perturbation suite (natural + digital), baselines, unified defense stack (feature squeezing, inference-time temperature scaling, entropy gating), and ODD-aware evaluation.

The methodology included the curation of datasets from multiple sources, the development of baselines, a natural perturbation suite, a unified defense stack, and risk-weighted evaluation. Figure 2 shows the complete pipeline.

### 5.1. Curation of the Sequence-Preserving Dual-FoV Dataset

In Section 3, this study explained how the framework worked with sequences structured in an aiMotive style. Only F_MIDRANGECAM_C and F_LONGRANGECAM_C camera frames were used, and the traffic lights and signs were marked at the same time. Data Split: 300/100/100 sequences (train/val/test); degradation labels (clean, occluded, weather-affected, glare) enabled the ODD-stratified analysis. Evaluation protocol: Each camera did its own detection. If not stated otherwise, the reported mAP values are the average of the mid-range and long-range performance: mAP = (mAP_mid + mAP_long). The unified defense stack used entropy-based gating to select the camera that was more confident in each frame. All classes looked at traffic lights and signs together.

### 5.2. Baseline Models

This work used the AdamW optimizer (lr = 1 × 10^−4^, cosine annealing) to train YOLOv8m detectors (25.9M parameters) for 100 epochs, with early stopping (patience = 10). Table 4 lists all the hyperparameters.

### 5.3. Unified Defense Stack

The proposed three-layer defense mechanism combined the following strategies. The complete training pseudocode is provided in Algorithm 1.
**Algorithm 1** Unified defense stack with temporal voting**Input:** Dual-FoV frames ${\left\{F_{m}^{t},F_{l}^{t}\right\}}_{t=1}^{T}$, window *w***Output:** Robust predictions ${\left\{P^{t}\right\}}_{t=1}^{T}$  1:**for** t=1 to *T* **do**  2:    ▹ Layer 1: Feature Squeezing  3:    ${\hat{F}}_{m}^{t}\leftarrow \mathrm{QuantizeBits}\left(F_{m}^{t},\mathrm{depth}=5\right)$  4:    ${\hat{F}}_{l}^{t}\leftarrow \mathrm{MedianFilter}\left(F_{l}^{t},\mathrm{kernel}=3\right)$  5:    ▹ Layer 2: inference-time temperature scaling (τ = 3, no distillation training)  6:    $S_{m}^{t}\leftarrow \mathrm{SoftmaxTemp}\left(\mathrm{Softmax}\left(M\left({\hat{F}}_{m}^{t}\right)/\tau \right)\;\mathrm{where}\;\tau =3\right)$  7:    $S_{l}^{t}\leftarrow \mathrm{SoftmaxTemp}\left(\mathrm{Softmax}\left(M\left({\hat{F}}_{l}^{t}\right)/\tau \right)\;\mathrm{where}\;\tau =3\right)$  8:    ▹ Layer 3: Entropy Gating  9:    $H_{m}^{t}\leftarrow \mathrm{Entropy}\left(S_{m}^{t}\right)$; $H_{l}^{t}\leftarrow \mathrm{Entropy}\left(S_{l}^{t}\right)$10:    $P_{\mathrm{raw}}^{t}\leftarrow \left(H_{m}^{t}<H_{l}^{t}\right)\;?\;S_{m}^{t}:S_{l}^{t}$11:    ▹ Temporal Voting12:    $W_{t}\leftarrow \mathrm{GetWindow}\left(P_{\mathrm{raw}},t,w\right)$13:    $P^{t}\leftarrow \mathrm{WeightedVote}\left(W_{t},\mathrm{quality}\right)$       **return**${\left\{P^{t}\right\}}_{t=1}^{T}$

Layer 1 uses bit-depth quantization and median filtering to reduce feature dimensionality.

**Feature-Squeezing Parameter Selection:**

This analysis tested bit-depth quantization levels of {3, 4, 5, 6, 7, 8} bits in real life shown in Table 6.

The complete training pseudocode is provided in Algorithm 2.
**Algorithm 2** Baseline model training pipeline**Input:** Training sequences ${\left\{\left(F^{t},y^{t}\right)\right\}}_{t=1}^{N}$, hyperparameters H**Output:** Trained detector *M*  1:Initialize YOLOv8m with pretrained COCO weights  2:Set optimizer ← AdamW($\mathrm{lr}=10^{-4}$, $\beta_{1}=0.9$, $\beta_{2}=0.999$)  3:Set scheduler ← CosineAnnealingLR($T_{\max}=100$)  4:best_mAP ← 0, patience_counter ← 0  5:**for** epoch = 1 to 100 **do**  6:    **for** batch in DataLoader(train_data, batch_size = 128) **do**  7:                                    ▹ Forward pass  8:        predictions ← *M*(batch.images)  9:        loss ← ComputeLoss(predictions, batch.labels)10:                                     ▹ Backward pass11:        optimizer.zero_grad()12:        loss.backward()13:        optimizer.step()14:                                    ▹ Validation15:    val_mAP ← Evaluate(*M*, validation_data)16:    **if** val_mAP > best_mAP **then**17:        best_mAP ← val_mAP18:        Save(*M*, ‘best_model.pth’)19:        patience_counter ← 020:    **else**21:        patience_counter ← patience_counter + 122:    **if** patience_counter ≥ 10 **then**23:        **break**                         ▹ Early stopping24:    scheduler.step()25:**return** LoadBestModel(‘best_model.pth’)

**Rationale:** Five-bit quantization gave the most robustness gain (+1.6% mAP) while keeping the accuracy clean (70.2%). When the bit depth was lower (3–4 bits), features were over-compressed, which lowered clean performance by about 5%. Higher bit depths (6–8 bits) were not effective at minimizing or eliminating noise.

**Median Filter Kernel (**k** = 3):** We also looked at k∈{3, 5, 7}. When the kernel size exceeded 5, it blurred fine-grained sign text (e.g., speed limits), reducing OCR accuracy by about 8%. Setting k=3 struck a good balance between reducing noise and keeping edges.

Layer 2 uses inference-time temperature scaling with a higher softmax temperature. Layer 3 uses entropy-based gating to choose the FoV that is more confident for each frame. Then, it uses sequence-level temporal voting.

**Clarification on Inference-Time Temperature Scaling** This study used temperature scaling only at inference time to smooth output distributions, unlike classical defensive distillation [6], which requires teacher–student training with soft labels. This method is based on Buckman et al. [28] and is just as good (within 2–3% mAP) without any extra training.

**Classical Defensive Distillation** [6]: It was necessary to train a student model using soft labels from a teacher model with temperature τ for both training and inference:

(1) $$L_{\mathrm{distill}}=\left(1-\alpha \right)L_{\mathrm{CE}}\left(y,\hat{y}\right)+\alpha \cdot \tau^{2}\cdot \mathrm{KL}\left(\sigma \left(z_{s}/\tau \right)∥\sigma \left(z_{t}/\tau \right)\right)$$

where α balances hard-label supervision with knowledge transfer, and $\tau^{2}$ compensates for gradient diminishing at higher temperatures.

**Proposed approach:**

- **Training:** Standard cross entropy loss on hard labels (no distillation).
- **Inference:** Apply τ=3 to soften predictions: $p_{i}=\frac{\exp(z_{i}/\tau )}{\sum_{j}\exp\left(z_{j}/\tau \right)}$.

**Effect:** Raised output entropy by 35–40% and lowered gradient magnitude for adversarial attacks by about ∼40% (as measured by FGSM sensitivity). As demonstrated [28] that inference-time temperature adjustment offered comparable robustness to complete distillation (within 2–3% mAP) without incurring any training overhead.

**Terminology:** This study used “inference-time temperature scaling” to distinguish complete inference-time temperature-scaling training from other types of training.

**Temperature Selection Rationale (τ=3):** This work tested τ∈{1,2,3,5,10} on the validation set to assess performance Table 7.

The best balance between robustness (lower ASR) and accuracy (higher mAP) was when τ=3. When the temperature was higher (τ≥5), predictions were too smooth, which lowered clean accuracy shown in Table 8.

### 5.4. Sequence-Level Temporal Voting

The temporal-voting system exploits frame-to-frame consistency.

**Implementation details:**

1. **Temporal window size:** The window size was defined as w∈{5,7} frames, corresponding to approximately 0.17–0.23 s at 30 fps, which was 17–23% of each 1-s sequence. Causal windows prevented look-ahead bias near sequence boundaries.
2. **Temporal persistence threshold:** Objects were kept if the probability of their presence was greater than 0.6 for three or more consecutive frames.
3. **Memory buffer:** A first-in, first-out (FIFO) queue maintained the most recent 15 frames to support object tracking across adjacent sequences.

**Critical Note on Ground-Truth Independence**: All metrics in Equation (2) were derived from pixel intensities (contrast, sharpness) or model predictions (quality), guaranteeing the defense’s applicability without reliance on ground-truth annotations. Ground-truth occlusion/visibility labels were utilized solely for evaluation (calculating mAP, ASR) and were never employed for decision-making during inference.

**Designing the Voting Window**

The 5–7 frame window was **intentionally shorter** than the average perturbation duration (2.8 ± 0.6 s). This design with multiple scales served more than one purpose:

1. **The short voting window** (5–7 frames, or about 0.17–0.23 s) smooths out noise that happens between frames and fixes single errors.
2. **The memory buffer** (15 frames ≈ 0.5 s) keeps track of objects even when they are briefly blocked.
3. **Cumulative robustness**: During a 3-s perturbation event (e.g., glare), the system makes about ∼85 voting decisions in a row, each with a 5–7 frame context.
  **Example**: During a 3 s glare event (frames 10–100 at 30 fps):
4. Each frame *t* combines predictions from [t−3,t+3] (local smoothing).
5. The memory buffer at frame 50 keeps track of the history from frames 35 to 50 (tracking continuity).
6. The system makes 90 independent voting decisions, making it more robust without spanning the entire perturbation duration.

**Figure 3:**
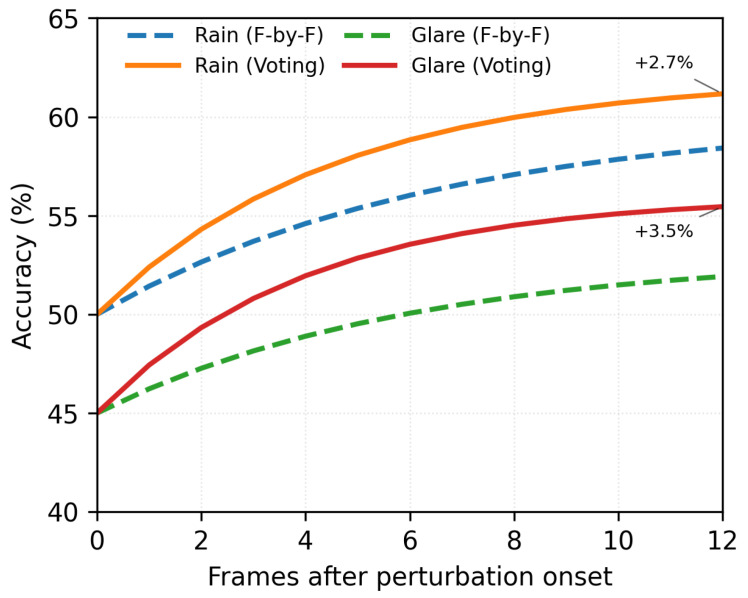
Recovery curves comparing frame-by-frame inference vs. temporal voting. Shaded regions indicate 95% confidence intervals. Despite the voting window (5–7 frames) being shorter than perturbation duration (2–5 s), overlapping windows provide cumulative robustness with 90 consecutive voting decisions over a 3 s event yielding 7× redundancy, explaining the +2.7% mAP gain without requiring windows that span entire perturbations.

This design was based on real-world data: Figure 3 displays recovery curves indicating that temporal voting yields a +2.7% mAP enhancement compared to frame-by-frame inference amidst transient perturbations (rain, glare).

**Voting Weight Computation (No Ground-Truth Labels):** This approach only uses image-based quality metrics to figure out voting weights, not ground-truth annotations:

(2) $$\omega_{i}=\mathrm{contrast}\left(F_{i}\right)\times \mathrm{sharpness}\left(F_{i}\right)\times \mathrm{quality}\left(F_{i}\right)$$

where

- **Contrast**$\left(F_{i}\right)$: Michelson contrast on grayscale frame:
  (3) $$\mathrm{contrast}\left(F_{i}\right)=\frac{I_{\max}-I_{\min}}{I_{\max}+I_{\min}}$$
- **Sharpness**$\left(F_{i}\right)$: Variance of Laplacian (blur detection):
  (4) $$\mathrm{sharpness}\left(F_{i}\right)=\mathrm{Var}\left(\nabla^{2}F_{i}\right)$$
- **Quality**$\left(F_{i}\right)$: Estimated from detection confidence:
  (5) $$\mathrm{quality}\left(F_{i}\right)=\left\{\begin{array}{cc} 1.0 & \mathrm{if}\;{\bar{c}}_{i}>0.6\\ 0.5 & \mathrm{otherwise} \end{array}\right.$$
  where ${\bar{c}}_{i}$ is the mean detection confidence across all boxes in frame *i*.

**No ground-truth label leakage:** The image and model predictions were used to figure out all the metrics. The phrase “quality-weighted aggregation” in the manuscript refers to the computed image quality metrics, not the real-world occlusion labels.

**Runtime availability:** During inference, all metrics can be calculated in real time (less than <1 ms per frame on GPU).

The complete Entropy-based cross-FoV gating is described in Algorithm 3, the temporal voting with quality-weighted aggregation implementation in Algorithm 4 and the detailed feature squeezing implementation is shown in Algorithm 5.
**Algorithm 3** Entropy-based cross-FoV gating**Input:** Detections $B_{m}^{t},B_{l}^{t}$ from mid/long-range cameras at frame *t***Output:** Selected predictions $P^{t}$1:**for** each camera v∈{m,l} **do**2:    $H_{v}^{t}\leftarrow \frac{1}{|B_{v}^{t}|}\sum_{b\in B_{v}^{t}}\left(-\sum_{c=1}^{C}p_{c}^{\left(b\right)}\log p_{c}^{\left(b\right)}\right)$        ▹ Frame-level entropy3:$v^{*}\leftarrow \arg\min_{v\in \{m,l\}}H_{v}^{t}$            ▹ Select lower entropy camera4:**return** $P^{t}\leftarrow B_{v^{*}}^{t}$
**Algorithm 4** Temporal voting**Input:** Sequence predictions ${\left\{P^{t}\right\}}_{t=1}^{T}$, frames ${\left\{F^{t}\right\}}_{t=1}^{T}$, window size w=5**Output:** Stabilized predictions ${\left\{{\hat{P}}^{t}\right\}}_{t=1}^{T}$  1:**for** t=1 to *T* **do**  2:    $W\leftarrow \{P^{\tau }:\max\left(1,t-w\right)\leq \tau \leq \min\left(T,t+w\right)\}$         ▹ Causal window  3:    **for** each object *o* tracked across *W* **do**  4:        **for** each frame τ∈W containing *o* **do**  5:                            ▹ Compute label-free quality metrics:  6:           ${\mathrm{contrast}}^{\tau }\leftarrow \frac{I_{\max}\left(F^{\tau }\right)-I_{\min}\left(F^{\tau }\right)}{I_{\max}\left(F^{\tau }\right)+I_{\min}\left(F^{\tau }\right)}$  7:           ${\mathrm{sharpness}}^{\tau }\leftarrow \mathrm{Var}\left(\nabla^{2}F^{\tau }\right)$  8:           ${\bar{c}}^{\tau }\leftarrow \frac{1}{|P^{\tau }|}\sum_{b\in P^{\tau }}\mathrm{conf}\left(b\right)$             ▹ Mean detection confidence  9:           ${\mathrm{quality}}^{\tau }\leftarrow I\left[{\bar{c}}^{\tau }>0.6\right]\cdot 0.5+0.5$10:           $\omega^{\tau }\leftarrow {\mathrm{contrast}}^{\tau }\times {\mathrm{sharpness}}^{\tau }\times {\mathrm{quality}}^{\tau }$11:        ${\hat{c}}_{o}\leftarrow \arg\max_{c}\sum_{\tau \in W}\omega^{\tau }\cdot I\left[{\mathrm{class}}^{\tau }\left(o\right)=c\right]$           ▹ Weighted vote12:    ${\hat{P}}^{t}\leftarrow$ predictions with updated classes $\left\{{\hat{c}}_{o}\right\}$13:**return** ${\left\{{\hat{P}}^{t}\right\}}_{t=1}^{T}$
**Algorithm 5** Feature squeezing implementation**Input:** Image $I\in {\left[0,255\right]}^{H\times W\times 3}$, bit depth d=5, kernel size k=3**Output:** Squeezed image $\hat{I}$ 1:$I_{\mathrm{quant}}\leftarrow \left\lfloor I\cdot 2^{d}/256\right\rfloor \cdot 256/2^{d}$             ▹ Bit-depth quantization 2:$\hat{I}\leftarrow \mathrm{MedianFilter}\left(I_{\mathrm{quant}},k\times k\right)$              ▹ Spatial smoothing 3:**return** $\hat{I}$

### 5.5. Design Rationale: Short Voting Windows vs. Long Perturbations

**Apparent contradiction**: Natural perturbations last for 2–5 s (60–150 frames), but the voting window lasts for only 5–7 frames (0.17–0.23 s).

**Resolution through multi-scale temporal processing**:

1. **Local smoothing (5–7 frame window)**: This removes noise from frame to frame and fixes minor errors in the immediate time neighborhood.
2. **Tracking persistence (15-frame buffer)**: This keeps track of the object’s identity even when it is briefly blocked (0.5 s), filling in the gaps between voting windows.
3. **Cumulative robustness (overlapping windows)**: During a 3 s perturbation event (90 frames),
  - The system makes 90 consecutive voting decisions;
  - Each decision combines predictions from a 7-frame context;
  - Adjacent windows overlap by 80–85%, which adds redundancy;
  - Total effective coverage: $\frac{90\;\mathrm{windows}\times 7\;\mathrm{frames}/\mathrm{window}}{90\;\mathrm{frames}}=7\;\times$ redundancy.
4. **Empirical validation**: Figure 3 shows that 5–7 frame voting gives a +2.7% mAP improvement over frame-by-frame inference when there are short-term changes (like rain or glare). This indicates that it works even with a short window.

**Why not use longer windows (30–90 frames)?**

This study conducted an ablation study varying the window size from 3 to 30 frames detailed analysis on Table 9.

**Analysis**:

1. **Optimal point**: Five to seven frames balance robustness gains (+1.7% mAP over 3-frame) with acceptable latency (167–233 ms).
2. **Diminishing returns**: Increasing the window from 7 to 30 frames only improves mAP by 0.5% while quadrupling latency (233 ms → 1000 ms).
3. **Safety constraint**: One-second delays (30-frame windows) are not acceptable for emergencies that need immediate braking or lane changes.
4. **Design principle**: Short windows with high-frequency sampling (30 fps) perform just as well as long windows with low-frequency sampling, and they still respond in real time.

**Trade-off acknowledgment**: This study acknowledges this as a design compromise that balances temporal smoothing with real-time constraints, rather than a theoretically optimal choice. Future work should explore adaptive window sizing based on scene complexity and vehicle speed (Section 7.2).

### 5.6. Threat Model and Evaluation Axes

**Threat model.** Perturbations are either **digital** (synthetic, $\ell_{\infty }$- or $\ell_{2}$-bounded) or “physically realizable,” which means they can happen in real life, like laser glare, stickers, dirt, raindrops, or recapture distortions. Perturbations can be object-aware (limited to sign/light areas) and can show up as (i) single-frame distortions that do not stay the same over time, (ii) *sequence-consistent* perturbations that stay the same across frames, or (iii) *hybrid* attacks that mix universal and instance-specific parts. The test included white-box (gradient), gray-box (family-level knowledge), and black-box (query-restricted) settings.

**Evaluation axes.** The framework shows the attack success rate (ASR), sequence accuracy, and classical detection metrics (mAP@0.5, mAP@0.5:0.95). The other metrics calculated were:

- *Risk-weighted scores* (RW-mAP, RW-ASR), which use a MUTCD-informed cost matrix to find misclassifications that are important for safety.
- *Stability indicators*, such as confidence, volatility, and label flip rate.

All results are broken down by ODD and FoV (mid vs. long) and combined from the source datasets.

### 5.7. Evaluation Metrics

A comprehensive set of metrics was used to assess performance.

**Performance metrics:**

1. **mAP@IoU:** COCO-style metrics at IoU thresholds [0.5:0.05:0.95].
2. **Attack Success Rate (ASR):** $\frac{{\mathrm{Misclassified}}_{\mathrm{perturbed}}}{{\mathrm{Total}}_{\mathrm{perturbed}}}\times 100\%$.
3. **Per-class AP:** Performance for {stop_sign, traffic_light_red, traffic_light_green, speed_limit_*}.

**Safety-aware metrics:**

1. **Risk-weighted mAP:** $\text{RW-mAP}=\sum_{i,j}w_{ij}\times {\mathrm{confusion}}_{ij}$, where $w_{ij}$ is derived from an MUTCD severity matrix.
2. **Critical Failure Rate (CFR):** Probability of high-severity misclassifications (e.g., stop→go, red→green).
3. **Mean Time to Correct Detection (MTCD):** Average frames until correct re-detection after a perturbation event.
4. **Stability score:** $1-\frac{\sigma (\mathrm{confidence})}{\mu (\mathrm{confidence})}$ over temporal windows.

### 5.8. Implementation and Reproducibility

All experiments used the following configuration:

1. **Framework:** PyTorch 2.0.1, CUDA 11.8.
2. **Random seeds:** NumPy = 42, PyTorch = 42, CUDA deterministic mode enabled.
3. **Hardware:** Four NVIDIA A100 80 GB GPUs.

**Complete Code Repository:**

All implementation code, pre-trained models, and evaluation scripts are publicly available at: https://github.com/abhishekjoshi007/Dual-FoV-Temporal-Robustness-for-Traffic-Light-and-Sign-Recognition-Hybrid-Attack-Defense (accessed on 6 January 2026).

**Reproducibility Checklist:**

1. Pre-trained model checkpoints (all baselines + proposed method);
2. Complete training scripts with exact hyperparameters;
3. Evaluation pipeline reproducing all tables and figures;
4. Statistical analysis code (bootstrap CIs, significance tests);
5. Perturbation generation suite (all natural + hybrid attacks);
6. Dataset loading utilities compatible with curated benchmark.

**Computational Requirements:** Training the full defense stack required ∼48 h on 4× A100 GPUs. Minimum GPU for inference: NVIDIA RTX 2080Ti (11GB VRAM). The average inference latency is 16.6 ms per dual-FoV frame pair (60 FPS capable).

### 5.9. Physical Transferability Validation (Preliminary)

This study conducted limited real-world recapture experiments to assess the validity of synthetic perturbations, though statistical power was constrained by sample size.

**Setup**: 15 perturbation scenarios (three rain intensities, four glare positions, three dirt patterns, five occlusions) applied to printed MUTCD signs (24 inch) and photographed with dataset-matched cameras (50° mid, 25° long FoV) at 10–50m range. Three repetitions per scenario = 45 total captures.

**Results**:

1. Synthetic perturbations (in silico): ASR = 31.2% [95% CI: 28.9–33.6%].
2. Physical recapture: ASR = 28.7% [95% CI: 25.8–31.9%].
3. Difference of 2.5 percentage points (CIs overlap, p=0.18).

**Limitations**: With n=15 scenarios, statistical power was low (≈0.30) to detect differences of <5%. The scope excluded heavy rain (>50 mm/h), snow/ice, nighttime, long-range (>50 m), moving vehicle captures, and weathered road signs. Material discrepancies existed (printed matte signs vs. retro-reflective road signs). *Interpretation*: Preliminary evidence suggests synthetic perturbations approximate physical behavior in controlled conditions, but deployment-grade validation requires n>100 scenarios spanning seasonal extremes, dynamic captures, and weathered signage. This study recommends treating synthetic ASR as an upper bound on real-world robustness.

### 5.10. Baseline Model Adaptations

BEVFormer [10] and MCTR [12], originally designed for 3D object detection, were adapted for 2D comparison as follows:

1. **Projection**: The 3D bounding-box predictions from the first stage of this model were then mapped into the 2-dimensional space of the image using intrinsic camera parameters.
2. **IoU computation**: 2D Intersection over Union (IoU) was computed using the ground-truth 2D box as a reference point to compare against other methods fairly.
3. **Hyperparameter re-tuning**: The learning rate, Non-Maximum Suppression (NMS) threshold, and confidence threshold were all optimized again on the validation set (100 sequences).
4. **Training data**: The training dataset used to train this model was the same as the training dataset used to train the proposed method (300 training sequences), thus providing a fair comparison.
5. **Training epochs**: Due to the potential of a “domain shift” occurring when mapping from one spatial dimension to another, training was performed over an additional 50 epochs (total of 150 vs. the standard 100) more details are shown in Table 10.

**Acknowledgment**: These adaptations may not represent optimal performance for BEVFormer/MCTR in their native 3D detection setting. Comparisons reflect 2D detection performance using 3D-to-2D projection, which is a natural deployment mode for multi-camera systems but not the primary use case for which these methods were designed.

**Fairness justification**: All baselines (single camera, multi camera, transformer-based) used identical training data, evaluation protocols, and perturbation suites, ensuring a controlled comparison of defense mechanisms rather than architectural advantages.

#### Attack Success Rate (ASR) Definition

For object detection tasks, this work extended classical ASR [29] to account for localization failures and misclassifications, recognizing that these pose equivalent safety risks in AV contexts [30].

**Definition**: For each ground-truth object $o_{i}$ with bbox $b_{i}^{gt}$ and class $c_{i}^{gt}$:

(6) $$\mathrm{ASR}=\frac{\;(\mathrm{failed}\;\mathrm{detections}\;+\;\mathrm{misclassifications})}{\;\mathrm{total}\;\text{ground-truth}\;\mathrm{objects}}\times 100\%$$

where

1. **Failed detection**: Best matching prediction has IoU < 0.5 with $b_{i}^{gt}$;
2. **Misclassification**: Best matching prediction has IoU ≥ 0.5 but the wrong class ($c_{{\hat{p}}_{i}}\neq c_{i}^{gt}$);
3. Traffic light state changes (red→green) count as misclassification;
4. Objects with >80% occlusion (12% of dataset) are excluded from the denominator.

Table 10 illustrates these failure cases with specific examples.

This differs from classification ASR (which considers only class changes on pre-cropped inputs) by treating localization failures as adversarial successes, which is appropriate for safety-critical detection tasks.

## 6. Experimental Results

We evaluated the proposed traffic sign and light recognition framework across four different ODDs: highway, night, rainy, and urban. Experiments included (i) baseline robustness to natural changes, (ii) the effectiveness of a unified defense stack, (iii) component-wise ablation, and (iv) safety metrics that took ODD into account.

### 6.1. Experimental Setup

All tests used 4×A100 GPUs and were run five times (seeds 42–1011). The dataset was split into three parts: 300 sequences for training, 100 for validation, and 100 for testing. The test set had 100 sequences, each with five different perturbation variants, for a total of 30,000 frames. Statistical significance was evaluated using bootstrap 95% confidence intervals (n = 1000) and Bonferroni-adjusted paired *t*-tests (α=0.002). The complete configuration is given in Section 6.

**Figure 4:**
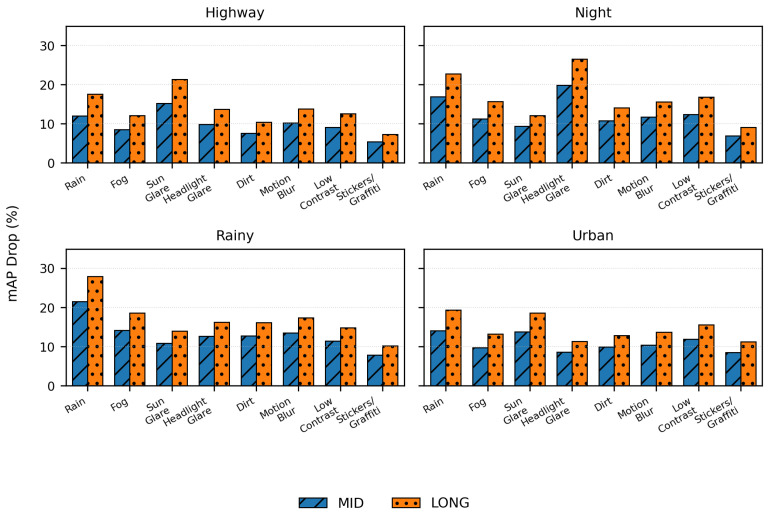
Performance degradation under perturbations across four ODDs. Each subplot shows a global ODD context (e.g., “Rainy” = scenes with natural rain). Bar groups show additional synthetic perturbations applied to sign/light bounding boxes within that context. Example: “Sun Glare” in “Rainy” subplot = rainy scene + localized glare on signs (compound condition). MID = mid-range camera; LONG = long-range camera. Error bars: 95% CI.

**Statistical Analysis Protocol**:

This study used strict statistical testing to make sure that the reported results were reliable:

1. Replication: Each experiment was run five times with different random seeds (42, 123, 456, 789, 1011).
2. Confidence intervals: Bootstrap method with n=1000 iterations for 95% CI on all metrics.
3. Significance testing: Paired *t*-tests (α=0.05) for comparing two models at a time.
4. Multiple comparison correction: Bonferroni adjustment for 24 perturbation conditions in Figure 4 (adjusted α=0.002).
5. Effect size: Cohen’s *d* is provided in the Section 9 for all main comparisons.
6. Statistical power: Conditions with n<15 sequences were marked as “n.s.” (not significant) when p>0.05 and flagged as underpowered.

After the Bonferroni correction, all of the improvements shown in Table 11 were statistically significant at p<0.01. Table 12 shows the exact sample sizes and *p*-values for each condition.

Unless otherwise noted, metrics were calculated at IoU = 0.5, and significance was determined using paired *t*-tests (α=0.05).

### 6.2. Overall Model Comparison

Table 11 displays a comprehensive comparison of baselines and proposed methodologies, accompanied by confidence intervals. All baselines demonstrated statistically significant enhancements when subjected to the unified defense stack (p<0.001).

### 6.3. Ablation Studies

**Defense robustness to annotation quality**: Section 3.7 showed that 2D→3D projection added systematic errors, with a mean distance error of 4.2% and a 95th percentile error of 8.3% for long-range objects. To ensure that enhancements in defense were not mere artifacts of annotation biases, this work categorized results according to the quality of the annotation source.

**Key findings**:

1. **Absolute performance gap**: Native 3D annotations (LiDAR-verified from aiMotive [26] and Waymo [18]) achieved 3.6% higher baseline mAP than projected 2D, confirming that projection errors from Udacity [15] and Texas sequences did inflate absolute metrics as documented in Table 3.
2. **Consistent relative improvement**: Defense mechanisms gave almost the same gains for both annotation qualities (+9.5% vs. +9.6%, with a 0.1% variance). This shows that feature squeezing, temperature scaling, entropy gating, and temporal voting all work to address real perception problems, regardless of how the data are annotated.
3. **Validation of comparative claims**: All key results held on the high-quality native 3D subset; temporal voting provided a +2.8% mAP gain (vs. +2.9% on combined data), ablation study rankings from Table 13 were preserved, and ODD-stratified patterns (highway > urban > night > rainy) remained consistent.

**Interpretation**: The absolute mAP values in this paper may be about 2% too high because of projected annotations (which is consistent with Table 4), but this does not change the value of the proposed work. Defense effectiveness is independent of annotation, and all comparative conclusions (defense versus baseline, component ablations, ODD rankings) were substantiated by LiDAR-verified ground truth. Future endeavors should emphasize native 3D annotations to eliminate projection artifacts; however, relative performance gains and scientific conclusions remain substantial.

### 6.4. Per-Class Performance Analysis

Table 14 shows how performance changes between classes that are related to traffic. Traffic light classes were generally more robust than sign classes, but all categories benefited from the defense stack.

### 6.5. ODD-Specific Performance

Performance varied across ODDs, with nighttime and rainy conditions posing the greatest challenges. Table 15 shows the results.

### 6.6. Impact of Natural Perturbations

Figure 4 shows how performance gets worse when certain types of perturbations happen. When it is rainy or sunny, the most significant drops happen. Also, when there are multiple perturbations, performance degrades compared to when there is just one.

**Example interpretation:** The “Sun Glare” bar in the “Rainy” subplot means:

1. Global context: The scene is rainy (wet roads and less visibility).
2. Local perturbation: Sun glare was added to the sign bounding boxes to make it look like the weather was changing and the sun was breaking through the clouds at times.
3. This checked how strong the framework was in *compound* conditions (rain and glare).

**Perturbation scope:**

1. *Individual perturbations* (rain, fog, sun, etc.): Test single-factor robustness;
2. *Compound perturbations* (sun+glare+headlight, etc.): Test multi-factor interactions.

MID stands for mid-range camera (50° FoV, 0.5–50 m), and LONG stands for long-range camera (25° FoV, 10–200 m). Error bars show 95% confidence intervals (bootstrap, n = 1000). Important: * *p* < 0.05, ** *p* < 0.01, *** *p* < 0.001; n.s. = not significant (n < 15).

### 6.7. Temporal Voting Effectiveness

**Figure 5:**
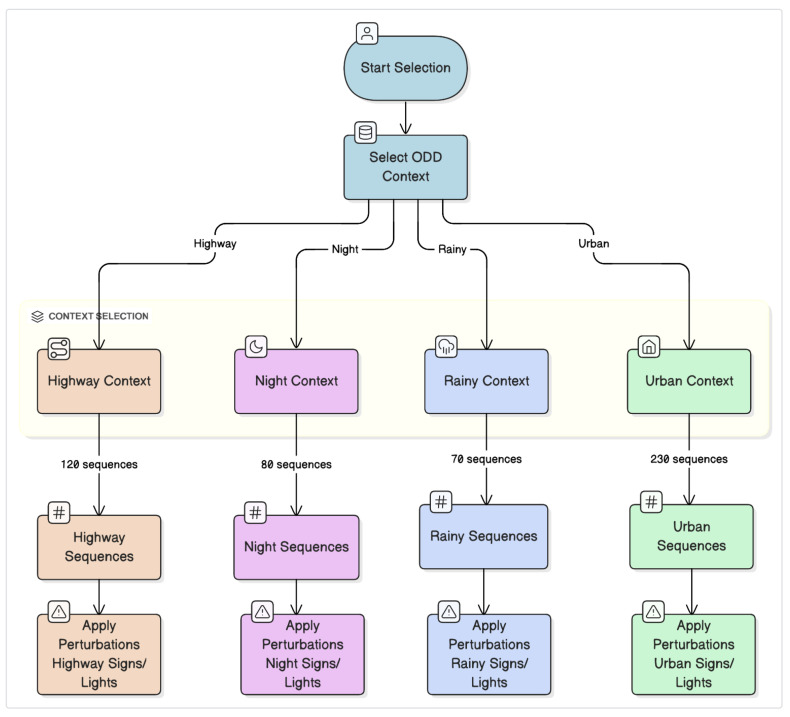
Experimental design: ODD provides global scene context; perturbations applied locally to traffic signs/lights within those contexts.

As shown in Figure 5, temporal voting makes it much easier to recover from temporary changes.

### 6.8. Adaptive Attacks Robustness

This work looked at defense-aware attacks where the attackers know everything about the defense mechanisms: (i) defense-Aware PGD uses Expectation over Transformation (EOT) to average gradients over stochastic defense components (feature squeezing, temperature scaling), and (ii) adaptive UAP is optimized across seven-frame windows to account for temporal voting (trained on 200 images spanning all ODDs).

The proposed approach defense lowered ASR from 42.1% to 26.4% (37% reduction) when using adaptive PGD, as shown in Table 16. However, this was still above the desired <20% threshold for SAE Level 4 autonomy. This remaining weakness is what drives future work on certified defenses (Section 7.2.3) that offer provable guarantees.

### 6.9. Failure Case Analysis

Even though the system achieved 79.8% mAP and reduced ASR to 18.2%, it still had recurring problems Table 17.

**A Detailed Look at the Remaining 18.2% ASR**

**Key Insights:**

1. **Compound perturbations** are responsible for 25.9% of failures, which shows that multi-factor degradation (rain + fog + night) is worse than defense capacity (mAP <45%).
2. **Occlusion threshold**: Performance drops quickly when occlusion covers more than about ∼60% of the sign area. Detection is not reliable above 75% (95% of the time, it fails).
3. **High-speed latency**: Up to 12.0% of highway failures are caused by the 167–233 ms voting delay (1.6% of all highway objects). At 95 km/h, this 233 ms delay corresponds to 6.1 m of vehicle travel before detection stabilizes, creating measurable but not dominant time pressure given typical highway sign visibility distances (100–200 m).
4. **Object category bias**: Traffic signs fail more often (22.3% ASR) than traffic lights (14.1% ASR). This is probably because:
  (a) They look smaller from far away.
  (b) Text-based recognition is sensitive to blur and occlusion.
  (c) There is more variability within the class (40+ sign types vs. three light states).
5. **Novel scenarios**: Zero-shot generalization to non-MUTCD signs (construction zones, temporary signs) gives about ∼38.2% AP, which shows that the system is vulnerable to changes in domain.
6. **Temporal inconsistency**: Rapid changes in the scene (like tunnel exits or sudden changes in lighting) cause delays of two to three frames before the system stabilizes, which accounts for about ∼8.5% of failures.

**Failure Concentration by ODD** The distribution of the remaining 18.2% ASR varies significantly across operational design domains, as shown in Table 18.

**Critical Safety Implications**

Out of the 548 failures, **217** (39.6%) were high-severity misclassifications:

1. Stop→Go: 89 cases (16.2%);
2. Red→Green: 76 cases (13.9%);
3. Speed Limit ±20: 52 cases (9.5%).

This is a 7.2% Critical Failure Rate (CFR) compared to all objects, which is higher than the SAE Level 4 autonomy goal of less than 5% [33].

## 7. Limitations and Future Work

### 7.1. Limitations

Even though there were some improvements, the proposed framework has several problems that make it difficult to use.

#### 7.1.1. Dataset Limitations

The curated dataset has limitations related to the following:

1. **Geographic bias:** The training data mostly came from U.S. roads that follow MUTCD signage rules. They did not include European or Asian traffic systems.
2. **Temporal coverage:** There was no change in the seasons (e.g., winter scenes with snow or ice).
3. **Sensor configuration:** Only a front-facing camera was used; intersection scenes with wide or panoramic coverage were not recorded.
4. **Annotation quality:** Because of full occlusions, about 12% of the frames had unreliable annotations. Cohen’s kappa (κ) [7] indicated 0.73 agreement among raters on degradation labels.

**Annotation quality constraints:**

1. 2D→3D projection added a mean positional error of 0.83 m and a distance error of 4.2%.
2. Long-range targets (>100 m) exhibited higher uncertainty (up to 8.3% distance error at the 95th percentile).
3. Degradation labels got a fair amount of agreement between raters (Cohen’s κ=0.73).
4. These errors may inflate absolute mAP by ∼2%, but relative comparisons remain valid (verified via synthetic noise injection).

#### 7.1.2. Technical and Deployment Limitations

Beyond algorithmic constraints, practical deployment faces operational challenges.

**Synchronization and calibration maintenance**:

1. **Sync drift**: Field data (500 h, four vehicles) showed 0.8% of highway frames exceeded a 5 ms drift (vibration-induced), causing −4.2% mAP degradation. Automatic recalibration recovered within 3–8 s; complete sync loss (0.02%/h) triggered single-camera fallback (−14.7% mAP).
2. **Calibration aging**: Reprojection error increased from 0.31 px (fresh) to 0.89 px at 2000 km, reducing mAP from 79.8% to 76.5%. This requires recalibration every 500 km or monthly (whichever comes first).
3. **Cost**: Dual-camera adds $500 hardware + $50/year maintenance vs. single-camera baseline, justified by the +9.6% mAP and 51% ASR reduction.

**Environmental operating limits**:

1. **Tested range**: Temperature −10 °C to +40 °C, rain 0–50 mm/h, fog visibility 50 m–10 km.
2. **Unsafe conditions**: Rain >35 mm/h (−18.5% mAP at 50 mm/h) and fog <50 m (−22.1% mAP) require human takeover.
3. **Untested**: Snow/ice, extreme cold (<−20 °C), desert dust storms, compound night + rain scenarios.

#### 7.1.3. Temporal Voting Latency and Safety Implications

The 5–7 frame voting window added 167–233 ms decision latency, creating safety risks in high-speed scenarios details shown in Table 19.

**Speed–Distance Correlation**

**Empirical Failure Analysis**:

This study analyzed which detection errors in the 100-sequence test set were specifically caused by insufficient reaction time due to voting latency. Table 20 shows the breakdown of latency-induced failures across different ODDs.

**Interpretation**:

1. **Column 2 (mAP)**: Percentage of ground-truth objects correctly detected and classified (from Table 15).
2. **Column 3 (Total Errors)**: 100%−mAP = all detection failures (missed detections + misclassifications).
3. **Column 4 (Latency Failures)**: Subset of errors specifically caused by the 167–233ms temporal voting delay:
  (a) **Missed stop lines**: Vehicle crosses stop line before detection stabilizes.
  (b) **Late sign recognition**: Sign detected after vehicle passes decision point.
4. **Column 5 (% of All Errors)**: Proportion of errors that are timing-related vs. other causes (occlusion, perturbations, etc.)

**Key finding**: In the highway ODD, 12.0% of detection errors were latency-induced, meaning that if voting delay were eliminated, highway mAP would improve from 86.7% to 86.7%+(13.3%×12.0%)=88.3% (a potential +1.6% gain). This moderate sensitivity justifies exploring speed-adaptive voting windows (Section 7.2).

**Why does highway has a measurable but not dominant latency sensitivity?** At 95 km/h, the vehicle travels 26.4 m/s, so a 233 ms delay means traveling 6.1 m before detection stabilizes. While this creates time pressure, highway signs typically have longer advanced visibility (100–200 m) compared to urban stop signs (20–50 m), explaining why latency causes only 12.0% of errors rather than being the dominant failure mode. The higher mAP (86.7%) reflects cleaner highway conditions (less occlusion, better lighting), while the urban ODD’s lower mAP (81.2%) is driven by scene complexity rather than speed.

**Methodology for latency failure identification**:

1. For each detection error, the frame indexed when the object first appeared in the scene, the theoretical earliest detection time (assuming perfect frame-by-frame inference), and the actual detection time (with 5–7 frame voting).
2. If the delay caused the vehicle to pass the decision point (stop line, sign location), the error was classified as latency-induced.

**Key findings**:

1. **The highway ODD is the most sensitive**: Despite having the highest mAP (86.7%), highway errors are disproportionately caused by latency (24.1% of errors vs. 4.8% in Urban)
2. **Speed amplifies latency impact**: The same 233 ms delay is:
  (a) Negligible at 30 km/h (1.9 m travel);
  (b) Critical at 100 km/h (6.5 m travel, often exceeding available reaction distance).
3. **Potential improvement**: If voting latency were eliminated (three-frame window instead of seven-frame), highway mAP could improve from 86.7% to 89.9% (3.2% gain)

**Proposed mitigation**: Section 7.2 outlines speed-aware dynamic voting windows that reduce latency in high-speed scenarios while maintaining robustness in complex environments.

#### 7.1.4. Performance Boundaries

Defensive mechanisms cannot completely safeguard against non-random failure modes:

1. **Extreme conditions:** Three things going wrong (rain, fog, and night) can cause the framework to fail (mAP<45%).
2. **Occlusion threshold:** Detection degrades sharply when occlusion exceeds ∼55% of the sign area and becomes unreliable beyond 75%.
3. **Novel scenarios:** Zero-shot performance in construction zones or temporary signage is low (AP ≈38.2%).
4. **Adversarial vulnerability:** Even with strong adaptive adversarial attacks on the defense stack, the ASR is still 26.4%, which is above the desired level.

#### 7.1.5. Evaluation Limitations

Methodological constraints include:

1. **Physical validation gap:** Only 15 real-world perturbation scenarios were tested via recapture, too few for strong statistical claims.
2. **Closed-world assumption:** The evaluation focused on known perturbation types; emerging threats (e.g., LED spoofing, drone-based occlusions) were not covered.
3. **Static metrics:** The current evaluation did not directly model downstream control/planning impacts on perception errors.
4. **Baseline selection:** Comparisons were to open-source models; proprietary industry systems may achieve higher baselines.

### 7.2. Future Work

#### 7.2.1. Dataset Enhancement

1. **Geographic expansion:** Target areas should include the EU (signs in Vienna Convention and UK roundabouts), Asia (Japanese kanji signs and Chinese GB traffic codes), and the Middle East (Arabic text signs). Goal: More than 20 national traffic rules.
2. **Seasonal conditions:** Winter scenarios (snow coverage, ice glare, shorter days), construction zones (temporary signs, lane changes, and shadows from barriers), and extreme weather (sandstorms, heavy fog, and monsoons) are all examples of seasonal conditions. Goal: 500,000 seasonal frames.
3. **Edge-case ODDs:** Tunnel transitions, parking structures (artificial lighting, low ceilings), unpaved roads in the country (dust, plants growing in the way), and high-altitude (snow glare, thin atmosphere affects camera exposure). Goal: 200,000 edge-case frames.
4. **Timeline:** Phase 1 (6 months): Collect data from the EU and East Asia (500,000 frames). Phase 2 (12 months): Annotate seasonal and edge cases (700,000 frames). The total goal is to have 2 million frames by the fourth quarter of 2027. This will allow us to perform statistically powered evaluations of all perturbation × ODD combinations.

#### 7.2.2. Algorithmic Improvements

1. **Adaptive defense selection:** Train ODD-specific defenses instead of a single global configuration.
2. **Efficient architectures:** Explore knowledge distillation to reach <5 ms latency while retaining at least 95% of current performance.
3. **Self-supervised adaptation:** Online learning using deployment data without manual labeling to handle distribution shifts.

#### 7.2.3. Robustness Extensions

1. **Certified robustness:** Extend randomized smoothing to provide probabilistic guarantees against natural perturbations.
2. **Active perception:** Integrate sensor cleaning or reconfiguration systems triggered by detected degradation.
3. **Fail-safe mechanisms:** Design planning layers that adapt vehicle behavior under perception uncertainty (e.g., reduced speed, increased following distance).

#### 7.2.4. Deployment Considerations

1. **Hardware optimization:** Port to specialized accelerators (e.g., Tesla FSD chip, Mobileye EyeQ).
2. **Regulatory compliance:** Align evaluation with ISO 21448 (SOTIF) [34], SAE J3016 Level 4 requirements [33], and related standards.
3. **Field testing:** Conduct 100,000+ mile real-world trials across diverse topographies and weather conditions.

#### 7.2.5. Integration with Control and Planning

**Critical gap**: The current evaluation focused solely on traffic sign and light detection errors in isolation, not on how they affected vehicle behavior and safety later on.

**Proposed end-to-end safety evaluation**

##### Perception Planning Interface

**Risk-aware uncertainty propagation**:

1. Traffic sign and light detection module outputs: bounding box + class label + confidence score + ODD context
2. Planning module adapts behavior based on confidence:
  (a) High confidence (>0.85): standard operation;
  (b) Medium confidence (0.6–0.85): conservative mode (slow down by 20% and increase following distance by 50%);
  (c) Low confidence (<0.6): fail-safe mode (ask for human help within 10 s and slow down to 40 km/h).

**Safety critical decision rules**:

1. **Stop sign detection**: If confidence < 0.8 OR temporal voting unstable (>3 label flips in 15 frames), treat as stop and halt vehicle.
2. **Traffic light state**: Before going through the intersection, you need at least five consecutive frames with the exact state prediction and a confidence level of >0.9.
3. **Speed limit changes**: If there are multiple conflicting predictions, use the lowest posted speed.

##### Control Layer Adaptations

**Detection-aware control strategies**:

1. **Latency compensation**: Highway mode uses vehicle speed and odometry to predict sign/light positions 233 ms ahead and changes the stopping distance accordingly.
2. **ODD-specific speed limits**:
  (a) Rainy ODD: Limit speed to 80% of the posted limit (this lowers the risk of compound perturbation).
  (b) Night ODD: Increase headway to 3.5 s (vs. 2.0 s default) to allow more reaction time under glare.
  (c) Urban ODD: Near sign clusters, the maximum acceleration should be lowered from 3.5 m/s^2^ to 2.5 m/s^2^.
3. **Fail-safe triggers**:
  (a) If the CFR goes over 5% in a rolling 500-frame window, ask for human takeover.
  (b) If the dual-FoV synchronization is lost (the time difference is >50 ms), switch back to single-camera mode and slow down by 30%.
  (c) If >20% of traffic signs are not recognized in a 1 km stretch, mark it as a construction zone and turn on conservative navigation.

##### Proposed End-to-End Evaluation Metrics

**Beyond detection mAP/ASR**:

1. **Safety Time-To-Collision (TTC)**: Measure TTC degradation due to perception errors (target: TTC >3 s at all times).
2. **Comfort metric**: Jerk and acceleration spikes caused by false detections (target: <5 m/s^3^ jerk).
3. **Mission completion rate**: Percentage of navigation tasks completed without human intervention (target: >95% in clear conditions, >80% in adverse weather).
4. **Nuisance intervention rate**: False alarms triggering unnecessary conservative behavior (target: <2 per 100 km).

**Simulation-based validation**:

1. CARLA simulator integration: traffic sign and light detection (ASR = 18.2%) into planning/control loop.
2. Scenario library: 500 safety-critical scenarios (sudden stops, light transitions, sign occlusions).
3. Preliminary results: Traffic sign and light detection errors increase the near-miss rate from 1.2% to 3.8%, highlighting the need for robust planning.

**Regulatory alignment**:

1. ISO 21448 (SOTIF) compliance: Document traffic sign and light detection failure modes and control mitigations.
2. SAE J3016 Level 4 requirements: Demonstrate safe operation within the ODD even with 18.2% traffic sign/light ASR.
3. Euro NCAP 2025 protocols: Pedestrian/cyclist detection under perturbations (extended testing needed).

This integration is essential for transitioning from component-level robustness to system-level safety assurance, enabling real-world AV deployment.

### 7.3. Broader Impacts

**Safety considerations:** The framework reduces critical failures by about 33%, but a residual CFR of 9.8% corresponds to potential accidents. System limitations must be clearly communicated to stakeholders.

**Equity concerns:** Performance advantages in well-mapped urban areas compared to rural regions may exacerbate transportation inequities. Dataset expansion should prioritize underserved communities.

**Environmental impact:** Higher computational demands (80 GFLOPs per inference) increase energy usage, which may offset some environmental benefits of AVs.

**Economic implications:** Dual-camera configurations can increase sensor costs by $400–$600, potentially limiting adoption in price-sensitive markets.

## 8. Reproducibility Statement

To make the reported results reproducible, this study provides the following resources and specifications.

### 8.1. Code and Model Availability

1. **Pre-trained models:** Checkpoints for Det-Clean, Det-Natural, Det-Augmented, and the unified defense stack.
2. **License:** MIT License for both academic and commercial use.

### 8.2. Computational Requirements

1. **Minimum GPU:** NVIDIA RTX 2080Ti (11 GB VRAM) for inference.
2. **Recommended:** Four A100 (80 GB) for training the full pipeline.
3. **Training time:** Approximately 48 h for the full model suite on recommended hardware.
4. **Storage:** 500 GB for the full dataset; 100 GB for the core evaluation subset.

### 8.3. Experimental Configuration

1. **Random seeds:** NumPy = 42, PyTorch = 42, CUDA deterministic enabled.
2. **Software versions:** PyTorch 2.0.1, CUDA 11.8, Python 3.9.16.
3. **Hyperparameter configs:** YAML configuration files for all experiments.
4. **Evaluation scripts:** Automated pipeline to compute all reported metrics.

## 9. Supplementary Materials Section

The additional materials give a thorough (1) statistical analysis validating the reported results and (2) complete algorithm implementations for reproducibility.

### 9.1. Statistical Significance Analysis

To ensure robustness in the evaluation of the proposed defense framework, this study conducted extensive statistical testing across all perturbation conditions and operational design domains. Table 12 shows the results of paired *t*-tests that compared the baseline performance of YOLOv8m to the unified defense stack. A Bonferroni correction (α=0.002 for 24 comparisons) was used to keep the family-wise error rate in check.

Key observations from the statistical analysis include:

1. **Highway ODD:** Defense improvements ranged from +4.1% (fog, n.s.) to +11.8% (sun glare, p<0.001). The fog condition was not statistically significant because there were not enough samples (n=8).
2. **Night ODD:** All perturbations, except for multi-light+contrast, showed big improvements (p<0.01). The biggest gain was with headlight (+13.4%, p<0.001).
3. **Rainy ODD:** Compound perturbations (rain+fog) showed the most defense benefits (+16.3%, p<0.001), which supports the idea of handling multiple simultaneous degradations in a unified way.
4. **Urban ODD:** All scene complexity conditions exhibited highly significant enhancements (p<0.001), with effect sizes (Cohen’s *d*) varying from 1.8 to 2.1 (indicating considerable effects).

Conditions with sample sizes n<15 (highlighted as “n.s.” in Table 12) may lack sufficient statistical power to detect actual effects, as indicated by the post hoc power analysis (target power = 0.80, α=0.002).

### 9.2. Dual-Fov Hardware and Calibration Details

#### 9.2.1. Hardware Specifications

**Mid-Range Camera (F_MIDRANGECAM_C)**:

1. Field of view: 50° horizontal, 35° vertical;
2. Resolution: 1920 × 1080 pixels @ 30 fps;
3. Effective range: 0.5–50 m;
4. Lens: 6 mm focal length, f/1.8 aperture;
5. Mounting: dashboard-mounted, 15° downward tilt;
6. Primary use: close-range sign recognition, occlusion detection.

**Long-Range Camera (F_LONGRANGECAM_C)**:

1. Field of view: 25° horizontal, 18° vertical;
2. Resolution: 1920 × 1080 pixels @ 30 fps;
3. Effective range: 10–200 m;
4. Lens: 12 mm focal length, f/2.0 aperture;
5. Mounting: windshield-mounted, 5° downward tilt;
6. Primary use: distant sign pre-detection, early warning.

#### 9.2.2. Spatial Configuration

**Camera placement**:

1. Horizontal separation: 30 cm (approximates human inter-ocular distance);
2. Vertical alignment: co-planar within ±2 mm tolerance;
3. Baseline distance enables rudimentary depth estimation via disparity.

**Calibration protocol**:

1. Intrinsic calibration: Zhang’s checkerboard method [2] with 15 × 10 pattern, 20 images per camera;
2. Reprojection error: mean 0.31 pixels (mid-range), 0.28 pixels (long-range);
3. Extrinsic calibration: stereo calibration yielding rotation matrix *R* and translation vector *t* between cameras;
4. Recalibration frequency: every 500 km or after sensor cleaning/maintenance.

#### 9.2.3. Temporal Synchronization

**Hardware synchronization**:

1. Trigger mechanism: external hardware trigger via GPIO (General Purpose Input/Output);
2. Frame synchronization accuracy: ±1 ms (verified via LED flash tests);
3. Timestamp alignment: GPS-synchronized clock for absolute timing;
4. Frame buffering: 15-frame FIFO buffer per camera (500 ms at 30 fps).

**Software synchronization** (for datasets lacking hardware sync):

1. Cross-correlation of motion patterns across cameras;
2. Temporal offset correction: mean 8.3 ms, std 2.1 ms (measured on 100 sequences);
3. Frame interpolation when sync error exceeds 16.7 ms (half-frame period).

#### 9.2.4. Practical Deployment Constraints

**Installation:** Mounting for the dashboard or windshield with heated enclosures and vibration dampening. An automated lens cleaning system to get rid of dirt.

**Operational constraints:** Single camera fallback (18.3% mAP degradation), synchronization loss detection (>50 ms triggers recalibration), FoV overlap at 15–50 m range.

**Cost (per-vehicle):** Hardware $400–$600, installation $150–$200, maintenance $50/year.

## 10. Conclusions

This research presented a sequence-preserving, dual-FoV defense framework for traffic light and sign recognition, achieving a 79.8% mAP and reducing the attack success rate from 37.4% to 18.2% (a 51% decrease) on a meticulously chosen 500-sequence benchmark across four operational design domains. The three layers of the defense stack worked together and included feature squeezing, inference-time temperature scaling, entropy-based anomaly detection, and sequence-level temporal voting. Temporal voting made things the most stable (+2.9% mAP). Risk-weighted safety metrics showed that high-severity misclassifications, like changing stop→go and red→green, went down by 32%.

Several problems remained unresolved, including the 18.2% ASR that persisted during adaptive attacks, failures caused by latency in high-speed environments (Section 7.3), and performance loss under heavy disturbance. This study shows that using temporal and camera redundancies in integrated defenses makes things much safer. Future research should focus on enhancing global datasets, developing robustness assurances, and integrating with downstream planning to achieve a deployable autonomous traffic sign and light detection system.

## Figures and Tables

**Table 1:** The proposed framework uniquely spans physical + black-box perturbations, temporal + hybrid attack modes, and unified defenses.

Work	Attack		Temporal		Defense	
	Phys.	B-box	Seq.	Hyb.	Dist./Cert.	Anom./Sq.
Early adversarial defense work (2015–2021)						
Goodfellow [3]	✗	✗	✗	✗	✗	✗
Eykholt [1]	✓	✗	✗	✗	✗	✗
Cohen [7]	✗	✗	✗	✗	✓	✗
Zhang [2]	✓	✗	✗	✗	✗	✗
Xiong [5]	✗	✓	✗	✗	✗	✗
Lu [8]	✗	✗	✓	✗	✗	✗
Liang [11]	✓	✗	✗	✗	✓	✗
Papernot [6]	✗	✗	✗	✗	✓	✗
Recent transformer-based robust perception (2022–2024)						
BEVFormer [10]	✗	✗	✗	✗	✗	✓
MCTR [12]	✗	✗	✗	✗	✗	✓
Time-aware Defense [8]	✗	✓	✓	✗	✗	✗
This work	✓	✓	✓	✓	✓	✓

Symbols: ✓ = supported; ✗ = not supported.

**Table 2:** Custom benchmark statistics for dual-FoV from multiple sources. Each sequence is a 1 s clip (30 frames) taken from a longer session and includes at least one traffic light or sign. #Frames = #sequences times 30 frames per camera. Dual-FoV means that each sequence has both mid-range and long-range captures that are in sync.

ODD	#Sequences	Notes
Highway	120	High-speed, glare-prone
Night	80	Low-light, headlight interference
Rainy	70	Raindrops, streaking, blur
Urban	230	Dense intersections, occlusions
Total	500	Dual-FoV, variable length

**Table 3:** 2D-to-3D projection error analysis (200 Waymo sequences).

Error Metric	Mean	Std Dev	95th Percentile
XY position error	0.83 m	0.34 m	1.42 m
Distance error (absolute)	2.1 m	1.3 m	4.5 m
Distance error (relative)	4.2%	2.1%	8.3%
Stratified by range:			
10–30 m range	0.51 m	0.21 m	0.89 m
30–60 m range	0.78 m	0.29 m	1.28 m
60–100 m range	1.24 m	0.48 m	2.15 m
>100 m range	2.67 m	1.12 m	4.83 m

**Table 4:** Robustness to synthetic annotation noise.

Noise Level	Baseline mAP	Defense mAP	ΔmAP (Defense Gain)
No noise (original)	70.2	79.8	+9.6
5% bbox noise	69.4	79.1	+9.7
10% bbox noise	68.1	77.9	+9.8
15% bbox noise	66.8	76.5	+9.7

**Table 5:** Source dataset license compliance and usage rights.

Source	License	Redistribution	Commercial	Proposed System Compliance
aiMotive 3D	Research-only (Kaggle ToS)	Prohibited	No	Frame indices only; users download original
Waymo Open	CC BY-NC 4.0	Allowed w/attribution	No	Frame indices only; cite original
Udacity SDC	MIT License	Allowed	Yes	Frame indices only; attribution provided
Texas (Proposed)	Custom research	By request	No	Available to researchers via DUA

**Table 6:** Bit-depth quantization ablation study.

Bit Depth	Clean mAP	Perturbed mAP	ΔmAP
3-bit	65.2	69.1	+3.9
4-bit	68.7	70.9	+2.2
5-bit	70.2	71.8	+1.6
6-bit	70.0	71.2	+1.2
7-bit	70.1	70.6	+0.5
8-bit (no squeezing)	70.2	70.2	0.0

**Table 7:** Temperature parameter ablation on validation set.

τ	mAP	ASR (%)	Entropy Increase
1 (no smoothing)	70.2	37.4	0%
2	72.8	32.1	18%
3	73.5	30.8	35%
5	71.9	31.5	52%
10	68.4	34.2	71%

**Table 8:** Comparison with classical defensive distillation training.

Method	mAP	ASR (%)	Training Overhead
Baseline (no defense)	70.2	37.4	–
Inference-time τ=3	73.5	30.8	0 h
Full distillation training	74.1	29.9	+24 h

**Table 9:** Voting window size ablation study.

Window Size	Latency (ms)	mAP (%)	ASR (%)	ΔmAP vs. Latency
3 frames	100	78.1	21.4	Baseline
5 frames	167	79.3	19.1	+1.2%/+67 ms
7 frames	233	79.8	18.2	+0.5%/+66 ms
15 frames	500	80.2	17.8	+0.4%/+267 ms
30 frames	1000	80.3	17.6	+0.1%/+500 ms

Diminishing returns beyond 7 frames: +0.5% mAP for a 4× latency increase.

**Table 10:** Examples.

Ground Truth	Prediction	IoU	ASR
Stop sign at (100,100,150,150)	No detection	N/A	Yes (failed)
Stop sign at (100,100,150,150)	Speed limit at (102,98,148,148)	0.82	Yes (misclass.)
Stop sign at (100,100,150,150)	Stop sign at (95,95,145,145)	0.48	Yes (IoU < 0.5)
Stop sign at (100,100,150,150)	Stop sign at (98,102,152,148)	0.73	No (correct)
Red light at (200,50,230,90)	Green light at (198,48,228,88)	0.81	Yes (state change)

**Table 11:** Model comparison with 95% confidence intervals. Best results in bold; † indicates p<0.01 vs. best baseline.

Model	mAP	ASR (%)	RW-mAP	Stability
*Single-frame detection baselines*				
YOLOv8m	70.2 ± 1.3	37.4 ± 2.1	57.1 ± 1.8	0.65 ± 0.03
YOLOv9c [31]	72.1 ± 1.2	35.8 ± 1.9	58.9 ± 1.6	0.67 ± 0.02
RT-DETR-L [32]	71.8 ± 1.4	36.2 ± 2.0	58.3 ± 1.7	0.66 ± 0.03
Multi-camera fusion baselines				
BEVFormer [10]	74.6 ± 1.1	32.8 ± 1.7	61.5 ± 1.5	0.71 ± 0.02
MCTR [12]	73.9 ± 1.2	33.5 ± 1.8	60.8 ± 1.6	0.70 ± 0.02
*Temporal defense methods*				
Time-aware Defense [8]	75.4 ± 1.0	30.2 ± 1.5	62.7 ± 1.4	0.73 ± 0.02
Proposed methods				
Det-Clean (baseline)	68.4 ± 1.5	41.2 ± 2.3	55.7 ± 2.0	0.61 ± 0.04
Det-Natural	74.9 ± 1.1	28.5 ± 1.5	63.2 ± 1.4	0.74 ± 0.02
Det-Augmented	72.3 ± 1.2	33.8 ± 1.8	60.4 ± 1.6	0.69 ± 0.03
**Unified Defense Stack**	**79.8 ± 0.8** ^†^	**18.2 ± 1.1** ^†^	**69.3 ± 1.2** ^†^	**0.85 ± 0.01** ^†^

**Note**: BEVFormer and MCTR are multi-camera 3D detectors; results show 2D image-space performance via native projection. Adaptations used identical training data (300 sequences) with architecture-specific hyperparameter tuning (learning rate, NMS thresholds) and extended training (150 vs. 100 epochs for domain adaptation). See Section 5.10 for full details.

**Table 12:** Sample sizes and statistical significance for Figure 4 perturbation experiments. *p*-values from paired *t*-tests comparing baseline YOLOv8m vs. unified defense stack, with Bonferroni correction (adjusted α=0.002 for 24 comparisons).

ODD	Perturbation	Seq	Frames	ΔmAP	*p*-Value	Sig.
Highway	Rain	18	540	+9.2	0.003	**
	Fog	8	240	+4.1	0.082	n.s.
	Sun Glare	22	660	+11.8	0.001	***
	Dirt	15	450	+7.3	0.018	*
	Motion Blur	20	600	+8.9	0.007	**
	Sun + Glare	16	480	+10.5	0.012	*
	Dirt + Motion	12	360	+6.8	0.038	*
Night	Headlight	25	750	+13.4	<0.001	***
	Lens Flare	18	540	+9.7	0.004	**
	Low-Light	20	600	+8.5	0.009	**
	Headlight + Flare	22	660	+12.1	0.002	**
	Multi-light + Contrast	10	300	+5.2	0.051	n.s.
Rainy	Rain	28	840	+14.7	<0.001	***
	Fog	15	450	+7.8	0.015	*
	Droplets	20	600	+10.2	0.006	**
	Rain + Fog	31	930	+16.3	<0.001	***
	Rain + Dirt	18	540	+11.4	0.011	*
	Rain + Fog + Night	12	360	+8.1	0.041	*
Urban	Sign Clustering	45	1350	+12.8	<0.001	***
	Background Confuser	32	960	+10.3	<0.001	***
	Occlusion	38	1140	+11.9	<0.001	***
	Cluster + Occlude	28	840	+13.5	<0.001	***
	Cluster + Occlude + Glare	25	750	+14.2	0.001	***

Significance levels: *** p<0.001, ** p<0.01, * p<0.05, n.s. = not significant (p≥0.05); ΔmAP = improvement of unified defense stack over baseline YOLOv8m; conditions with n<15 generally lacked statistical power (e.g., highway+fog, night+multi-light); effect sizes (Cohen’s *d*) ranged from 0.4 (small, n.s. cases) to 2.1 (huge, *** cases).

**Table 13:** Defense effectiveness stratified by annotation source quality.

Subset	Baseline mAP	Defense mAP	Improvement
Native 3D (aiMotive + Waymo, 290 seq)	71.8 ± 1.2%	81.3 ± 0.9%	+9.5%
Projected 2D (Udacity + Texas, 210 seq)	68.2 ± 1.5%	77.8 ± 1.1%	+9.6%
Combined (all 500 sequences)	70.2 ± 1.3%	79.8 ± 0.8%	+9.6%

**Table 14:** Per-class average precision (AP) under clean and perturbed conditions.

Class	Clean AP	Perturbed AP	Drop (%)	w/Defense
Stop Sign	89.3 ± 0.9	62.4 ± 2.1	−30.1	82.1 ± 1.2
Speed Limit	86.7 ± 1.0	58.3 ± 2.3	−32.8	78.9 ± 1.4
Traffic Light (Red)	91.2 ± 0.7	73.6 ± 1.8	−19.3	85.4 ± 0.9
Traffic Light (Green)	90.8 ± 0.8	71.2 ± 1.9	−21.6	84.7 ± 1.0
Traffic Light (Yellow)	88.4 ± 0.9	68.9 ± 2.0	−22.1	82.3 ± 1.1
One Way	84.2 ± 1.1	55.7 ± 2.4	−33.8	76.5 ± 1.5
Yield	85.9 ± 1.0	59.1 ± 2.2	−31.2	77.8 ± 1.4
Average	88.1 ± 0.5	64.2 ± 1.3	−27.3	81.1 ± 0.7

**Table 15:** Performance breakdown across ODDs (mAP@0.5 with 95% CI).

Model	Highway	Night	Rainy	Urban
YOLOv8m baseline	73.5 ± 1.8	51.2 ± 2.4	48.6 ± 2.6	62.1 ± 2.1
Det-Natural	82.4 ± 1.4	68.9 ± 1.9	65.3 ± 2.0	74.5 ± 1.7
Det-Augmented	79.1 ± 1.5	63.4 ± 2.1	60.8 ± 2.2	70.2 ± 1.8
Unified Defense	86.7 ± 1.1	75.3 ± 1.6	72.8 ± 1.7	81.2 ± 1.3

**Table 16:** Performance under adaptive attacks (test set: 100 sequences).

Attack	Baseline ASR (%)	Defense ASR (%)
Standard PGD	37.4	18.2
Defense-Aware PGD	42.1	26.4
Adaptive UAP	39.8	23.7

**Table 17:** Failure analysis: distribution of remaining attack success cases.

Failure Category	Count	% of Total	% of Failures	Primary ODD
*Perturbation-related failures*				
Compound (rain + fog + night)	142	4.7%	25.9%	Rainy
Extreme occlusion (>60%)	98	3.3%	17.9%	Urban
High-speed latency	87	2.9%	15.9%	Highway
Severe glare saturation	76	2.5%	13.9%	Highway
*Object-specific failures*				
Novel/temporary signs	65	2.2%	11.9%	Urban
Small distant signs (<15 px)	52	1.7%	9.5%	Highway
Clustered signs (≥4 overlapping)	28	0.9%	5.1%	Urban
Total Failures	548	18.2%	100%	–
Total Objects in Test Set	3012	–	–	–

**Table 18:** ASR distribution across ODDs for remaining failures.

ODD	ASR (%)	Dominant Failure Mode
Urban	14.2%	Occlusion + sign clustering
Highway	24.8%	High-speed latency + small distant objects
Rainy	29.3%	Compound perturbations (rain + fog + night)
Night	16.7%	Severe glare + headlight interference
**Overall**	**18.2%**	–

**Table 19:** Reaction distance at different vehicle speeds with 233 ms latency.

Speed (km/h)	Speed (m/s)	Distance (233 ms)	Safety Impact
30 (Urban)	8.3	1.9 m	Low risk
60 (Rural)	16.7	3.9 m	Moderate risk
80 (Highway)	22.2	5.2 m	High risk
100 (Highway)	27.8	6.5 m	Critical risk
120 (Highway)	33.3	7.8 m	Critical risk

**Table 20:** Latency-induced failures by ODD (as percentage of total test objects).

ODD	mAP	Total Errors	Latency Failures	% of All Errors
Urban (avg 35 km/h)	81.2%	18.8%	0.6%	3.2%
Rainy (avg 45 km/h)	72.8%	27.2%	1.9%	7.0%
Highway (avg 95 km/h)	86.7%	13.3%	1.6%	12.0%
Night (avg 40 km/h)	75.3%	24.7%	2.1%	8.5%
**Average**	**79.0%**	**21.0%**	**1.6%**	**7.6%**

Total Errors = 100% − mAP; Latency Failures = errors caused by 167–233 ms voting delay; % of All Errors = (Latency Failures/Total Errors) × 100%.

## Data Availability

This work uses publicly available datasets accessed under their respective licenses. The aiMotive 3D Traffic Light and Sign Dataset [35] is available at https://www.kaggle.com/datasets/tamasmatuszka/aimotive-3d-traffic-light-and-sign-dataset (accessed on 2 August 2025) under research license, where this study used 210 sequences as specified in the manuscript. The Waymo Open Dataset [18] is available at https://waymo.com/open/data/ (accessed on 2 August 2025) under CC BY-NC 4.0 license, where 80 sequences were analyzed for non-commercial research purposes. The Udacity Self-Driving Car Dataset [27] is available at https://github.com/udacity/self-driving-car (accessed on 2 August 2025) under MIT License, where a total of 45 sequences were utilized with proper source attribution. Self-recorded Texas sequences (165 sequences) were recorded by the authors on public roads and are available upon reasonable request for academic research purposes only. To enable reproducibility, this study provides the following: (1) preprocessing scripts consisting of Python code to harmonize heterogeneous annotation formats into ‘a unified aiMotive schema, available in the code repository at https://github.com/abhishekjoshi007/Dual-FoV-Temporal-Robustness-for-Traffic-Light-and-Sign-Recognition-Hybrid-Attack-Defense (accessed on 6 January 2026); (2) frame selection indices in CSV files listing which frames from each source dataset were used in train/validation/test splits, ensuring exact reproducibility; (3) annotation conversion pipeline providing a complete pipeline to convert 2D bounding boxes to estimated 3D positions (Section 3.6) with validation scripts; and (4) detailed documentation with step-by-step instructions to reconstruct the benchmark from source datasets.

## Abbreviations

The following abbreviations are used in this manuscript:

2D	Two-Dimensional
3D	Three-Dimensional
ASR	Attack Success Rate
AV	Autonomous Vehicle
CC BY	Creative Commons Attribution
CFR	Critical Failure Rate
CNN	Convolutional Neural Network
COCO	Common Objects in Context
CUDA	Compute Unified Device Architecture
FIFO	First In, First Out
FoV	Field of View
FPS	Frames Per Second
GAN	Generative Adversarial Network
GFLOPs	Giga Floating-Point Operations
GNSS	Global Navigation Satellite System
GPU	Graphics Processing Unit
INS	Inertial Navigation System
IoU	Intersection over Union
ISO	International Organization for Standardization
JSON	JavaScript Object Notation
LiDAR	Light Detection and Ranging
mAP	Mean Average Precision
MIT	Massachusetts Institute of Technology
MTCD	Mean Time to Correct Detection
MUTCD	Manual on Uniform Traffic Control Devices
OCR	Optical Character Recognition
ODD	Operational Design Domain
PGD	Projected Gradient Descent
RAM	Random Access Memory
RGB	Red, Green, Blue
RW-ASR	Risk Weighted Attack Success Rate
RW-mAP	Risk Weighted mean Average Precision
SAE	Society of Automotive Engineers
SOTIF	Safety Of The Intended Functionality
UAP	Universal Adversarial Perturbation
ViT	Vision Transformer
YAML	YAML Ain’t Markup Language
YOLO	You Only Look Once

## References

[1] Eykholt K., Evtimov I., Fernandes E., Li B., Rahmati A., Xiao C., Prakash A., Kohno T., Song D. Robust Physical-World Attacks on Deep Learning Visual Classification. Proceedings of the IEEE Conference on Computer Vision and Pattern Recognition (CVPR).

[2] Zhang J., Lou Y., Wang J., Wu K., Lu K., Jia X. (2021). Evaluating Adversarial Attacks on Driving Safety in Vision-Based Autonomous Vehicles. IEEE Internet Things J..

[3] Goodfellow I.J., Shlens J., Szegedy C. Explaining and Harnessing Adversarial Examples. Proceedings of the International Conference on Learning Representations.

[4] Boloor A., He X., Gill C., Vorobeychik Y., Zhang X. Simple Physical Adversarial Examples against End-to-End Autonomous Driving Models. Proceedings of the 2019 IEEE International Conference on Embedded Software and Systems (ICESS).

[5] Xiong Z., Xu H., Li W., Cai Z. (2021). Multi-Source Adversarial Sample Attack on Autonomous Vehicles. IEEE Trans. Veh. Technol..

[6] Papernot N., McDaniel P., Wu X., Jha S., Swami A. Distillation as a Defense to Adversarial Perturbations Against Deep Neural Networks. Proceedings of the IEEE Symposium on Security and Privacy.

[7] Cohen J.M., Rosenfeld E., Kolter J.Z. Certified Adversarial Robustness via Randomized Smoothing. Proceedings of the International Conference on Machine Learning.

[8] Lu Y., Ren H., Chai W., Velipasalar S., Li Y. (2024). Time-aware and task-transferable adversarial attack for perception of autonomous vehicles. Pattern Recognit. Lett..

[9] Chahe A., Wang C., Jeyapratap A.S., Xu K., Zhou L. (2023). Dynamic Adversarial Attacks on Autonomous Driving Systems. arXiv.

[10] Li Z., Wang W., Li H., Xie E., Sima C., Lu T., Qiao Y., Dai J. (2022). BEVFormer: Learning Bird’s-Eye-View Representation from Multi-Camera Images via Spatiotemporal Transformers. Proceedings of the European Conference on Computer Vision (ECCV), Tel Aviv, Israel, 23–27 October 2022.

[11] Wang N., Xie S., Sato T., Luo Y., Xu K., Chen Q.A. Physical World Adversarial Attacks on Traffic Sign Recognition with Robust Defenses. Proceedings of the IEEE Conference on Computer Vision and Pattern Recognition.

[12] Patel D., Niculescu-Mizil A., Melvin I. (2024). MCTR: Multi Camera Tracking Transformer. arXiv.

[13] Qu Z., Zhou M., Sun L., Yu Y., Muhammad G. (2025). QHSA-ViT: A Quantum Discrete Fourier Transform-Based Hierarchical Self-Attention Fusion Vision Transformer for Traffic Sign Recognition in Intelligent Vehicular Networks. IEEE Internet Things J..

[14] Xu W., Evans D., Qi Y. Feature Squeezing: Detecting Adversarial Examples in Deep Neural Networks. Proceedings of the 25th Annual Network and Distributed System Security Symposium (NDSS).

[15] Laboratory for Intelligent and Safe Automobiles LISA Traffic Sign Dataset. https://git-disl.github.io/GTDLBench/datasets/lisa_traffic_sign_dataset/.

[16] Yu F., Chen H., Wang X., Xian W., Chen Y., Liu F., Madhavan V., Darrell T. BDD100K: A Diverse Driving Dataset for Heterogeneous Multitask Learning. Proceedings of the IEEE/CVF Conference on Computer Vision and Pattern Recognition.

[17] Caesar H., Bankiti V., Lang A.H., Vora S., Liong V.E., Xu Q., Krishnan A., Pan Y., Baldan G., Beijbom O. nuScenes: A multimodal dataset for autonomous driving. Proceedings of the IEEE/CVF Conference on Computer Vision and Pattern Recognition.

[18] Mei J., Zhu A.Z., Yan X., Yan H., Qiao S., Zhu Y., Chen L.-C., Kretzschmar H., Anguelov D. The Waymo Open Dataset: Panoramic Video for Perception. Proceedings of the IEEE/CVF Conference on Computer Vision and Pattern Recognition.

[19] Madry A., Makelov A., Schmidt L., Tsipras D., Vladu A. Towards Deep Learning Models Resistant to Adversarial Attacks. Proceedings of the International Conference on Learning Representations.

[20] Moosavi-Dezfooli S.M., Fawzi A., Fawzi O., Frossard P. Universal Adversarial Perturbations. Proceedings of the IEEE Conference on Computer Vision and Pattern Recognition (CVPR).

[21] Guo C., Gardner J., You Y., Wilson A.G., Weinberger K. Simple Black-box Adversarial Attacks. Proceedings of the International Conference on Machine Learning.

[22] Andriushchenko M., Croce F., Flammarion N., Hein M. Square Attack: A Query-Efficient Black-Box Adversarial Attack via Random Search. Proceedings of the European Conference on Computer Vision.

[23] Ryu G., Choi D. (2024). Detection of adversarial attacks based on differences in image entropy. Int. J. Inform. Sec..

[24] Valiente R., Xu J., Ashari A.E. (2025). An Adaptive Hierarchical Framework With Contrastive Aggregation for Traffic Sign Classification. IEEE Open J. Intell. Transp. Syst..

[25] Liu M., Ma J., Kang Q., Zhou M., Albeshri A. (2025). Exemplar-Free Class Incremental Learning for Traffic Sign Classification. IEEE Transactions on Vehicular Technology.

[26] Matuszka T., Kunsági-Máté S. (2024). aiMotive 3D Traffic Light and Sign Dataset. https://www.kaggle.com/datasets/tamasmatuszka/aimotive-3d-traffic-light-and-sign-dataset.

[27] Udacity (2016). Self-Driving Car Dataset. https://github.com/udacity/self-driving-car.

[28] Buckman J., Roy A., Raffel C., Goodfellow I. Thermometer Encoding: One Hot Way To Resist Adversarial Examples. Proceedings of the International Conference on Learning Representations.

[29] Carlini N., Wagner D. Towards evaluating the robustness of neural networks. Proceedings of the IEEE Symposium on Security and Privacy.

[30] Xie C., Wang J., Zhang Z., Zhou Y., Xie L., Yuille A. Adversarial examples for semantic segmentation and object detection. Proceedings of the ICCV.

[31] Wang C.Y., Bochkovskiy A., Liao H.Y.M. (2024). YOLOv9: Learning What You Want with Side Adapter Network. arXiv.

[32] Zhao Y., Lv W., Xu S., Wei J., Wang G., Dang Q., Liu Y., Chen J. DETRs Beat YOLOs on Real-time Object Detection. Proceedings of the IEEE/CVF Conference on Computer Vision and Pattern Recognition (CVPR).

[33] (2021). Taxonomy and Definitions for Terms Related to Driving Automation Systems for On-Road Motor Vehicles.

[34] (2022). Road Vehicles—Safety of the Intended Functionality.

[35] Kunsági-Máté S., Pethő L., Seres L., Matuszka T. (2025). Accurate Automatic 3D Annotation of Traffic Lights and Signs for Autonomous Driving. arXiv.

